# Patient and Parent Views on a Web 2.0 Diabetes Portal—the Management Tool, the Generator, and the Gatekeeper: Qualitative Study

**DOI:** 10.2196/jmir.1267

**Published:** 2010-05-28

**Authors:** Sam Nordfeldt, Lena Hanberger, Carina Berterö

**Affiliations:** ^4^Division of Nursing SciencesDepartment of Medical and Health SciencesLinköping UniversityLinköpingSweden; ^3^Division of Child and Adolescent PsychiatryDepartment of Clinical and Experimental MedicineLinköping UniversityLinköpingSweden; ^2^Division of PediatricsDepartment of Clinical and Experimental MedicineLinköping UniversityLinköpingSweden; ^1^Center for Medical Technology AssessmentDepartment of Medicine and Health SciencesLinköping UniversityLinköpingSweden

**Keywords:** Web 2.0, eHealth, childhood chronic disease, type 1 diabetes, self-care, disease management, patient information, apomediation, networking, social media, learning, health care professionals, children, adolescents, parents

## Abstract

**Background:**

The Internet has undergone rapid development, with significant impact on social life and on modes of communication. Modern management of type 1 diabetes requires that patients have access to continuous support and learning opportunities. Although Web 2.0 resources can provide this support, few pediatric clinics offer it as part of routine diabetes care.

**Objective:**

We aimed to explore patients’ and parents’ attitudes toward a local Web 2.0 portal tailored to young patients with type 1 diabetes and their parents, with social networking tools such as message boards and blogs, locally produced self-care and treatment information, and interactive pedagogic devices. Opportunities and obstacles to the implementation of Web 2.0 applications in clinical practice were sought.

**Methods:**

Participants were 16 mothers, 3 fathers, and 5 young patients (ages 11-18 years; median 14 years) who each wrote an essay on their experience using the portal, irrespective of frequency and/or their success in using it. Two main guiding questions were asked. A qualitative content analysis was conducted of the essays as a whole.

**Results:**

Three main categories of portal users’ attitudes were found; we named them “the management tool,” “the generator,” and “the gatekeeper.” One category was related to the management tool functionality of the portal, and a wide range of concrete examples was found regarding useful facts and updates. Being enabled to search when necessary and find reliable information provided by local clinicians was regarded as a great advantage, facilitating a feeling of security and being in control. Finding answers to difficult-to-ask questions, questions portal users did not know they had before, and questions focusing on sensitive areas such as anxiety and fear, was also an important feature. A second category was related to the generator function in that visiting the portal could generate more information than expected, which could lead to increased use. Active message boards and chat rooms were found to have great value for enhancing mediation of third party peer-to-peer information. A certain level of active users from peer families and visible signs of their activity were considered necessary to attract returning users. A third category was related to the gatekeeper function of the password requirement, which created various access problems. This and other unsuccessful experiences caused users to drop the portal. A largely open portal was suggested to enhance use by those associated with the child with diabetes, such as school personnel, relatives, friends and others, and also by young users somewhat unwilling to self-identify with the disease.

**Conclusions:**

Web 2.0 services have great potential for supporting parents and patients with type 1 diabetes by enhancing their information retrieval and disease management. Well-developed services, such as this one, may generate continued use and should, therefore, be carefully maintained and updated by health care professionals who are alert and active on the site with new information and updates. Login procedures should be simple and minimized as much as possible. The education of clinical practitioners regarding the use of Web 2.0 resources needs more attention.

## Introduction

The management of diabetes and other chronic diseases is based on the interplay between initiatives and resources on the part of patients, relatives, and health care professionals [1]. Modern pediatric diabetes treatment supports patients in gradually becoming their own treatment experts, and thus the balance in shared responsibilities is shifting over time to patients and their families [2]. This requires families to continue learning and to keep updated regarding treatment, self-care, and scientific findings. In recent decades, many pediatric diabetes practitioners have made efforts to enhance peer-to-peer support and learning with activities such as group education, evening meetings, parent groups, camps for adolescents, mailing list discussion groups, and chat rooms [3-7]. Meanwhile, information technology has undergone rapid development impacting significantly on social life and modes of communication [8]. Technical advances provide a foundation for proactive health systems that use information from multiple sources for support aimed improved health and avoidance of health risks [9]. These networked systems are increasingly connected to the world around them often through the use of portable devices, such as laptops and cell phones. Web 2.0 is an umbrella term describing a range of new collaborative Internet applications [9]. Compared to the earlier Web 1.0, Web 2.0 allows increased user participation in developing and managing content; this has changed the nature and value of the information [10]. This has resulted in dramatic changes in possibilities for informal and self-directed information seeking by individuals, implying that the individual is in command of what information should be sought and why it is important. A continuously greater proportion of online health-related information is created and maintained by apomediation from individuals other than health professionals, such as other patients [9]. Moreover, new criteria are being developed for evaluating the quality of medical advice [11]. Case studies of young people with a chronic health problem have found that young people are enthusiastic about “one stop shopping” sites that target them and their needs. Such sites might include focused chat rooms and message boards, for example [12].

The eHealth resolution WHA58.28, approved in 2005 by the World Health Assembly, stresses the importance of eHealth [13]. The resolution urges member states to make a range of efforts to develop eHealth services for all health sectors and create long-term strategic plans for development and specific implementation, such as reaching communities and vulnerable groups with services appropriate to their needs [13]. There is no doubt that such efforts are relevant for the care of children and adolescents with chronic disease. Interest in searching for health-related information online has been found to be greater among young people with type 1 diabetes and their families than in the general population [14]. Modern treatment of type 1 diabetes includes individualized education, intense multiple-dose treatment regimens, active self-control, and new insulin and insulin delivery technologies [15-17]. Nevertheless, a large proportion of young patients are still at risk for acute and/or long-term complications [18-20]. According to young Swedish patients with diabetes and their parents, improvements are needed regarding patient information and access to services [21]. For adult patients with diabetes, Internet-based interventions may improve access to health services, patient education, and quality of care, and have also been reported to influence these patients’ health care utilization, behavior, attitudes, knowledge, skills, and, to some extent, metabolic control [22-26]. For example, adult patients with diabetes have been perceived online support groups as helpful in improving coping strategies [27]. Interestingly, patients with poor metabolic control, and those with greater use of health care services, higher motivation, and/or less experience with diabetes treatment seem to benefit more than others from the use of electronic communication [28]. Improved quality of life has been reported, but overall, there has been little focus on patient perspectives in clinical studies [28].

However, clinical implementation of Internet-based support systems for young patients with type 1 diabetes seems to be a slow process compared with the rapid technological developments. While young patients frequently connect to various online networks, few of their health professionals are presently familiar with the rapidly emerging social network applications on the Internet. We found few Web 2.0 systems in routine use in pediatric diabetes care that have been developed and evaluated in collaboration with diabetic children and their families, although some studies have suggested potential benefits [7,29-32].

Proactive development of Web 2.0 applications including modern pedagogic devices has received little attention as compared with resources spent on care of late complications of diabetes. The Linkoping Diabit study, which served as a case study in this paper, is a bottom-up project run by practitioners and clinical researchers. Recently, a number of positive attitudes among practitioners involved in the development of the Diabit Web 2.0 portal were reported [33]. The present study highlights views and voices on the portal from a sample of young patients with diabetes and their parents.

The aim of this study was to explore patients’ and parents’ attitudes toward a local Web 2.0 portal tailored to young patients with type 1 diabetes and their parents. Opportunities and obstacles to the implementation of Web 2.0 applications in clinical practice were sought.

## Methods

### Process of Care

In Sweden, all children and adolescents with diabetes are treated by hospital-based pediatric diabetes teams consisting of nurses and nurse specialists, physicians and dieticians, social workers, and/or clinical psychologists. The practitioners meet the young patients, along with their parents, when hospitalized at onset, and continue to see them as outpatients over many years. The process of care, the treatment policy, and perceived quality of care have been described elsewhere [16,21].

### Web 2.0 Portal

During the spring of 2006 the research group and the two participating diabetes teams launched an Internet portal named Diabit for invited patients and parents. This portal contained specific diabetes-related information and social networking functions such as message boards and blogs. The portal had been gradually developed from a previous design model, and a prototype was piloted in 2005 [32-34]. The user-centered design process for the portal and its contents included iterative sessions conducted over a long period of time with groups of patients and parents as well as with the involved diabetes teams.

The content of the portal was designed to be used by children, parents, and their practitioners who belonged to the respective local patient communities of the two hospitals. For younger children, the portal was targeted at their parents, and for children 12 years of age and over, the portal invited both parents and adolescents, based on adolescents’ growing responsibility for their treatment along with their increasing maturity and need of autonomy. For the community areas in the portal, there was a set of rules for use based on common sense and national laws, with individual users responsible for the information provided.

**Figure 1:**
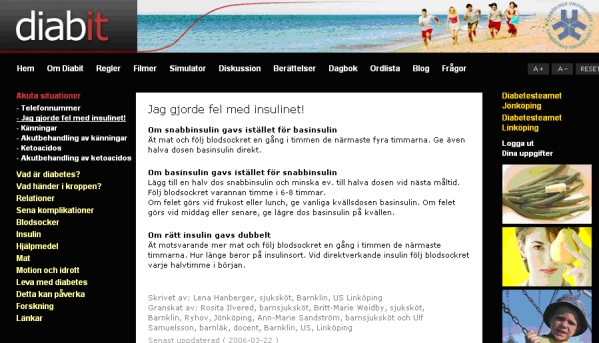
A sample of practitioners’ information, entitled “I made a mistake with the insulin!” with advice for specific emergencies.

The portal also included extensive text pages, education videos and online simulation software, which has been described elsewhere [31]. The practitioners’ information was based on scientific evidence and best clinical practice and aimed at creating a trustworthy and reliable source of information. Specific diabetes-related information on 13 main topics, divided into 99 subtopics/web pages had been written by an author group consisting of a nurse, a physician, and a dietician (Figure 1). The text at the bottom of the screen in Figure 1 shows the names of those who wrote and revised the webpage (giving names and affiliations), as well as the date of the last update.

As a next step, each section was discussed, revised, and cosigned by independent multiprofessional groups from the two hospitals. The portal also provided various services for medical prescription renewal, making appointments, sending questions, viewing questions and answers, contact information, photos of staff, and other general information about the local diabetes teams and their services. In addition, each respective group of professionals comprising the two local diabetes teams summarized important basic information using a personal tone when expressing, “What I may say to newly diagnosed children and their parents.”

### Study Population and Data Collection

The participants were parents and pediatric patients treated by diabetes teams at two pediatric clinics situated in southeastern Sweden that treat populations in their catchment areas of approximately 200 and 250 patients, respectively, below 19 years of age. During a clinical intervention study from 2006 to 2008, patients aged 12 to 18 years and mothers and fathers of all 510 patients were offered a personal password to the portal. Excluded were 18 families that declined to participate as well as 8 families that had been transferred to other centers. Thus, 484 invitation letters with one reminder were sent in September 2008 by mail (and, when possible, by email) asking recipients about their experiences using the Web portal, Diabit. Recipients could choose to respond either by email or by mail to an independent researcher who was not their practitioner. Of the 24 individuals who responded, 16 were mothers, 3 were fathers, and 5 were young patients aged 11 to 18 years old (median 14 years).

In the letter of invitation, patients and their parents were asked to write an essay about their positive as well as negative experiences using the portal, irrespective of frequency of use and/or success in doing so. Two leading questions were asked, followed by clarifying questions. These were: (1) Tell me about a situation when you succeeded in using Diabit. Has Diabit made managing the disease easier in any way? Are there any advantages in using Diabit? (2) Tell me about a situation when you did not succeed in using Diabit. Has Diabit become an obstacle in some way? Are there any disadvantages in using Diabit.

### Nonusers

Of the 24 respondents, 4 reported not having used the portal. The essays of one father and one mother who shared their reasons for nonuse were included, as well as the essay of a nonuser mother reflecting on user experiences of others and perceived needs. One young respondent was excluded who simply stated,

boy I don’t have any experience at all with Diabit, I haven’t used it at all

Thus, the respondents consisted of 23 persons. 

### Analysis

Considering the explorative aim of the study, the respondents’ essays were analyzed as a whole using techniques of conventional qualitative content analysis [35,36]. Qualitative content analysis can be applied to transcribed interviews, texts, narratives, letters, documents, protocols, and media, for example [37].

The analyses were performed by two of the authors (SN and CB), one of whom (CB) maintained a supervisory role throughout the study and was experienced in the methods used for data collection and analysis as well as in nursing and nursing research. The first author (SN) was experienced in clinical research, care of the target group, and the design and elements of the Web portal system.

Before beginning the analyses, both of these authors read all the primary data. As the text material was read, statements with similarities were clustered and summarized into tentative positive and negative categories based on the questions asked. The tentative categories with all respective statements were reviewed in detail. Unclear statements were explored with respect to the original context. Through iterative in-depth discussions, SN and CB recategorized the statements in a stepwise fashion, and a more logical and complete structure gradually emerged. After completion of this process, the material was put aside for six weeks of complete time-out. After this time-out, the complete sentences from the original text material were again reviewed in their original context and condensed into final categories by the same two authors, and final adjustments were made. Thus all the categories were validated through systematic repeated reviews of the material. To confirm and illustrate the categories and subcategories selected, quotations related to the respective categories were selected during the categorization process. The quotes used in the results section were selected to illustrate themes emerging from all respondents’ statements. 

Thus, the essays were analyzed and categorized by SN, and CB read and scrutinized the texts and the categories as well. For reliability, comparisons were made between SN’s and CB’s categorizing. Any discrepancies were resolved through discussion; no measure of interrater reliability was used. The risk of bias due to the authors’ preconceptions or expectations was prevented as far as possible through the repeated validations of the primary data and the in-depth review sessions.

### Ethics

The study was approved by the Research Ethics Committee of the Faculty of Health Sciences at Linköping University, Linköping, Sweden.

## Results

Three main categories of attitudes were identified/constructed during the analysis and given the names “the management tool,” “the generator,” and “the gatekeeper.” As shown in Table 1 each main category included a number of subcategories at varying levels of abstraction, and the categories were linked together by the underlying meanings [35].

**Table 1:** Overview of categories and subcategories

The Management Tool	The Generator	The Gatekeeper
Being enabled to find useful information and services	More than expected	Issues with passwords
Impact of diabetes experience and the disease trajectory	Peer-to-peer communication	Identity and stigmatization issues
Information available whenever needed or “just in time”		
Information perceived as reliable		
Availability of help in spite of limited resources of the local care teams		
Reasons for dropping the portal and suggestions to increase use		

### The Management Tool

As is the case when driving a car or running a complicated machine, it is necessary to manage the process of treatment and self-care of a long-term disease. To manage a process, certain tools are usually needed, some of which may be related to adequate and reliable information. With respect to diabetes treatment and self-care, reliable online information tools might be of value, and access to the tools at the time when the information is actually needed may increase their usefulness.

A set of positive attitudes toward the portal was presented by users of different ages and with different durations of diabetes experience. The portal was experienced as a well organized website, where it was easy to find one’s way around. Some respondents succeeded in logging onto Diabit every time they tried to do so, and these respondents stated that the portal was a good, reliable place to find current information and facts. Respondents also expressed that Diabit was valuable and that further use would be worthwhile. A parent of a newly diagnosed boy wrote:

father I’ve succeeded in using Diabit every time I’ve tried. I think it’s good with very interesting information.

Young Diabit users wrote:

girl I think the website is good and it’s good that there are a lot of facts.

girl I think Diabit is a good and simple site. It’s easy, smooth, cool, and really interesting to log onto Diabit.

#### Being Enabled to Find Useful Information and Services

Being enabled to succeed in a search for specific information seemed to be valuable both in general terms and regarding specific issues. The respondents reported a wide range of individual experiences concerning useful facts and updates on food, carbohydrates, the significance of fat, exercise, glycosylated hemoglobin and blood glucose, locally prescribed devices, the function of glucose meters in very cold weather, and current research in the field of diabetes. Furthermore, respondents also reported that it was helpful to be enabled, when needed, to make online blood glucose diagrams, to find contact information and information about the local staff, information about dealing with errors in insulin doses that had just occurred, and about insulin treatment when afflicted with acute gastroenteritis. Being able to find answers to difficult-to-ask questions, to find answers to questions they did not know they had before, and to find answers to questions focusing on sensitive areas such as anxiety and fear were also described by respondents as important functions of great benefit.

mother As a parent you need to read and hear about the importance of carbohydrates, the importance of fat, and so on, many times...

mother When my daughter felt mentally unwell last fall, the website was of great help to me since I could find information about anxiety and diabetes.

mother I think it’s important to be able to log onto the site and read it yourself without needing to ask questions that might be difficult to ask.

Thus, there appeared to be a great need for general and specific information about diabetes and self-care. Users’ success in retrieving problem-based information might facilitate being in control and coping with the disease in daily life.

mother Since there was no diabetes in our family, we were poorly informed about the disease. We’ve found quite a lot of useful information about diabetes on Diabit, both my husband and me….Thanks to that information and being able to surf around and read about different issues, everything from alcohol to exercise, trips, etc, we’ve gotten much better control.

mother There’s so much you want to get answers to….Diabetes is a tough disease especially in the teenage years, both for children and adults.

#### Impact of Diabetes Experience and the Disease Trajectory

The actual need may vary according to previous diabetes experience and other factors. The portal appeared to be of particularly great advantage for the more recently diagnosed young patients and their families. Some respondents reported greater use during the period shortly after onset of the disease, and others suggested that the portal was of great interest for those who were newly diagnosed.

mother I think we would have used Diabit a lot if he had gotten his disease now.

mother Diabit worked well with information about diabetes, especially in the beginning when we were searching for a lot of information.

On the other hand, the experience of already being in control and having felt secure with the treatment over a long period of time was one reason for limited use of the portal. Previous good contact with the practitioners, good continuity over time regarding such relationships, sufficient personal experience with living with diabetes, and perceived long-term success regarding treatment were mentioned as factors that might contribute to a low perceived need for repetitive use of the portal.

mother When my son was diagnosed with diabetes, I had had the disease myself since the age of 19, and I therefore already had a lot of knowledge about it…I think the website is well done and informative; it’s just that I often have the knowledge and experience needed to solve different problems or plan for different situations, which means that I seldom visit the site…

mother We logged onto Diabit in the beginning…Because things have gone so well for him, it hasn’t felt like there was a need to visit Diabit anymore. It happens less and less. If any questions arise, we might do so, but we’re in close contact with the doctor and nurse so maybe we turn first of all to them.

#### Information Available Whenever Needed or “Just in Time”

Being enabled as a parent or young patient to search for facts at the time they are needed, thus to find answers to current questions and to keep updated with the flow of information was regarded as a useful feature and as making life with diabetes easier. Logging onto Diabit from any computer and being able to get tips and advice, that is, getting theoretical information transformed into practical action, was appreciated and considered to be of great value.

mother Diabit makes it easy if you want to find an answer to some question you have. I see only advantages in using Diabit.

mother I think it’s great that there’s a search engine you can use if you want answers about diabetes….Everything’s gotten easier, I think it’s positive….Everything about Diabit is positive, I often log on and check what’s there.

girl Diabit makes it easy for me to quickly find answers to certain questions. I can log on at school if I need to. I haven’t failed to log onto Diabit. It’s easy to log on.

#### Information Perceived as Reliable

Respondents expressed a feeling of security in knowing that information and facts found on the portal had been checked by the local care teams. Thus, being enabled to find correct, reliable information provided by local practitioners was regarded as very advantageous, making it easier to feel secure and in control.

mother A big advantage with Diabit is that you can feel sure that the information you read there is correct. There’s naturally a huge amount of information on the Internet, but you can never be sure that there’s a reliable source for everything you read.

mother I think Diabit has been good since we know the facts and the information have been checked by those who are treating him.

#### Availability of Help in Spite of Limited Resources of the Local Care Teams

Respondents assumed that in light of limited access to the practitioners, the portal could be especially useful when help was needed.

mother …it has been and is hard to get in touch with the diabetes nurse. They’re very busy and there’s nowhere to email, only voicemail that works poorly. So if you need help I think you have definite problems. Then Diabit is certainly helpful.

#### Reasons for Dropping the Portal and Suggestions to Increase Use

Various unsuccessful user experiences, such as few hits from a specific search or seeing that there had been little activity in the practitioners’ news and updates sections of the portal, could create the perception that the practitioners were not “on their toes” (ie, alert to new developments and updating the information on the portal in a timely fashion). This could cause the user to drop the portal.

mother On the other hand it feels like not much “happens” on that website, I mean it’s not updated often enough…Of course it depends on the users, but the updates also have to do with other issues.

Individual respondents also expressed experiencing that the portal was valuable and that further use would be worthwhile. One respondent suggested using the portal to prepare for clinic visits, such as by filling in a form online with treatment updates and issues that the clinician should be aware of.

father I have no idea whether or not the website is visited a lot. But it’s absolutely worth more visits and more activity…A possible suggestion is that before the visit each individual could write his or her “report” on the Internet, prescriptions that are needed, and questions that might be asked, so that if possible, the diabetes nurse or the doctor can be prepared. Maybe a simple questionnaire?

Regular use of reminders was suggested to enhance use of the portal in the context of clinical care, and the view was expressed that the technical format of emailed newsletters should be kept simple.

mother A tip for you might be to send a reminder at regular intervals about the website’s existence or when there’s some news to read. As a user it’s a matter of seeing that you benefit from the website, but also that you are in the habit of regularly going out on the site and reading. I really and truly believe that this website has an important function.

mother Can’t you simply send the newsletter and other things as regular text, this way I only get a nice picture and a jumble of meaningless letters. You don’t need a lot of fancy formats, the information is what you should get.

### The Generator

A generator can mean many things and have many functions. One function is to convert one form of energy to another, for example, electric current that can be used for different needs. Visiting the portal may generate more information retrieval than initially planned, which may lead to increased use. In addition to benefits described in the previous categories, the interactive sharing of information mediated by patients and parents themselves seemed to be of particularly important value.

#### More Than Expected

A successful experience in using the portal could generate positive feelings and contribute to immediately extending use, to a new plan for continued use, and to a new desire to use the portal again. Such unplanned expansions of information retrieval could even include relevant issues about which the user might not yet have formulated any specific questions.

mother ...I sought information about something and didn’t find anything about just that, but a lot of other useful things…for example, information about those questions you don’t think of asking yourself, since you don’t know anything about those things.

A young Diabit user wrote:

girl The last time I was out on the Internet I wondered a little about how things were going in research. I thought that I’d log onto Diabit and check. Once I was there, I sat almost an hour and read different texts and even other things. I’m not that ”old” yet but have always liked to read and am interested in my diabetes and do as good a job as I can. I thought it was really interesting, and time just flew.

#### Peer-to-peer Communication

Regarding Web 2.0 services, active message boards and chat rooms were considered of great value for enhancing mediation of peer-to-peer information. Parents who had been enabled to share experiences with others online found it advantageous. One respondent with extensive personal experience with diabetes said that peer-to-peer communication between parents was a necessary element for the success of the portal.

mother I thought it was good to be able to read about the experiences of others. We aren’t alone in this.

mother Maybe Diabit can be something good, but that requires parents to give advice to parents.

Parents thought that an active message board and chat room would be beneficial, since being able to share experiences with others in “the same situation” was considered positive. A young respondent expressed a clear view on the need for a more functional chat room:

girl I want a better chat room on Diabit; it’s hard to understand what to do and how chatting works.

Respondents were of the opinion that to attract returning users, it was necessary to have a certain number of active users from peer families and visible signs of their activity on the message boards.

mother I checked the message board a few times when it was new, but it hadn’t gotten started much yet.

mother I hope the forum page really gets going with many users, since it’s good to be able to share experiences with others in the same situation.

### The Gatekeeper

A gatekeeper is someone or something that guards or monitors passage through a gate for some reason, for instance, by restricting or sometimes facilitating a flow of knowledge and information. A gatekeeper may sometimes also deny and, therefore, prevent entrance to some people. 

The major theme from reported negative experiences regarding the portal was related to various problems caused by restricted access. Thus it appeared that the effects of technical password procedures had the function of a gatekeeper.

#### Issues With Passwords

Password procedures, that is, having the key to the gatekeeper, appeared to limit the number of new users as well to limit access to the portal for some returning users. A gatekeeper effect from login procedures was created both by preexisting personal attitudes of users and by incidents of personal mismatch with use of the current login system. Such unsuccessful experiences might have led to discontinued use.

mother I think Diabit is valuable for those who have diabetes. But having to use a password is complicated…

mother We haven’t used Diabit very much; the main reason for that has been that you have to have a password.

Procedures for replacing lost passwords could create problems, and standard registration procedures with the option “create your own password” could also do so.

mother The problem I experienced was when a password was needed to log onto the website. I happened to lose my password and had problems getting a new one.

girl I logged onto the website one time and then tried to change my password. The next time I wanted to visit, neither my new password nor my old password worked. Since then I haven’t logged onto the site again.

In addition, information about logging onto the website that practitioners gave to newly diagnosed patients was sometimes delayed and even contradictory, with the result that the potential user gave up and dropped the idea of using the portal.

mother …it took a while to get a password for the website.

A largely open portal was suggested in order to make access easier for all patients and professionals, not only for practitioners, but also for school personnel. An open portal would facilitate its being used by others related to the child with diabetes, such as relatives and friends.

mother Also good in that you can recommend that school personnel can log onto Diabit and search for information and support concerning how they can handle their support of children with diabetes.

#### Identity and Stigmatization Issues

Users with particularly negative feelings about their disease and/or health care experiences might not be willing to go through the procedure for logging onto a disease-specific portal. Some respondents expressed thoughts about children or adults being forced to participate in something like a disease fellowship. Some ambiguity was expressed concerning this issue; identifying oneself as a person with diabetes was sometimes seen as beneficial and sometimes seen as detrimental.

mother I haven’t bothered logging onto Diabit since I’m quite ”care-injured” after many years in diabetes care with all that has involved in terms of a lack of integrity, meddling, ignorance, and an inability to see the whole patient.

mother …my son has refused to use it at all. He doesn’t want to identify himself through his disease at all and thinks it’s embarrassing and hard to be on a disease-related website

Based on individual experiences, to some respondents, an open portal seemed to be more in accord with the view that there are no secrets about having diabetes and that the disease is no one’s fault. The view they expressed was that an open portal might lead to more frequent use by people somewhat unwilling to identify with the disease; that is, if the portal were more open, then the issue of identification would be downplayed.

mother There aren’t really any secrets, right? It isn’t anyone’s fault that you have the disease…

mother I have a little trouble with the fact that you have to log onto the website as a whole, and I should think it would be enough if only certain parts required a login. For example, this would make it easier for my son if he got a sudden whim and wanted to have a quick look at the website, and it could also be good for relatives or friends who seek information.

## Discussion

This study confirms the well-known need for information related to childhood type 1 diabetes [1,21]. Variation exists over the course of the disease and between individuals in the early stages of the disease as well as in individuals’ approaches to daily self-care, and a long-term perspective to supporting these individuals and their varying needs is required [2,19,20,28]. At a time when young people are increasingly using computers and cell phones to connect to networks all over the world, health care practitioners and their service providers need to be alert to new developments in health services and health information for young people with diabetes and their parents.

### The Management Tool

As previously reported, young patients and their parents value being able to search for specific information when needed [14]. Adolescents and parents have expressed a wide range of specific needs that can be well understood and verified from a clinical perspective [1,5,6]. Findings from the current study as well as earlier studies suggest that enhanced online access to information might contribute to improved coping with the disease and increased control [28].

Regarding previously discussed security and trust issues [11,38], the perceived reliability of the information contained on the portal indicates its advantages for both patients and professionals. Reported positive experiences of finding reliable information may be due to the fact that local practitioners have been responsible for the portal, which contains specific articles written by the practitioners that are signed and personally updated by them (Figure 1). As long as patients and parents feel secure about the information available through the portal, successful management of diabetes is likely to be enhanced. Practitioners, on their part, are able to control the information they provide and refer to it in their practice whenever needed [33].

Although the Web 2.0 portal was found to be of great value in diabetes management for parents and patients, the portal did not appear to be easily handled by involved practitioners. And if users perceive that information is updated too infrequently, or if users are unsuccessful in finding the information they need, use of the service may decline. As has been previously pointed out, more attention needs to be paid to the education of clinical practitioners and others involved in the management of childhood chronic diseases using Web 2.0 resources for parents and patients [33,39,40].

### The Generator

Successful use of the portal appeared to generate more information retrieval than planned by the users; some spent more time on the site, and some found more information than expected or found other useful information and returned to the portal more often. Patients’ and parents’ accumulated experiences from everyday life form a knowledge base from which information can be shared with others [9]. Active message boards and chat rooms are of great value, as has been reported previously by young people with HIV [12]. Parents may inform other parents about a wide range of issues, as can adolescents and children at their own level based on their maturity and autonomy. Younger children may use occasional chatting simply for enjoyment and to identify with others with diabetes, as it may be difficult to find peers with diabetes in their physical neighborhoods. The opportunity for interaction with others distinguishes the Web 2.0 portal in this study from traditional computer-based education efforts related to diabetes or other chronic disease. With evolving Web 2.0 technology, traditional authorities are increasingly being replaced by apomediaries, which are tools or peers that lead to trustworthy information or add credibility to information [9,10].

Thus it appears from the data that the apomediated information and support from peers in a Web 2.0 system is of added and unique value, and that this type of information and support cannot be replaced by practitioners’ information per se. In order to attract users and make use of the generator effect, a living site is needed that incorporates social media such as active message boards, chats and blogs, as well as frequent news and updates from practitioners [33]. Some subjects’ perceptions of a low level of activity on the portal reflect that initially the portal was open only for selected patients in a clinical study and that practitioners were not used to the new technology. Health care administrators and stakeholders should focus on expanding clinical practitioners’ use of interactive Web 2.0 services for improved care and support of people with long-term diseases [33].

### The Gatekeeper

A factor that limited spontaneous and active use of the portal was the login requirement for access. Many negative experiences with logging onto the portal were expressed by the respondents, ranging from problems with practitioners’ distribution of preprepared passwords to their newly diagnosed patients to the automatic and manual procedures for replacing lost passwords. Indeed, the most commonly reported negative experiences were related to various problems accessing the portal. It appears that the effects of technical password procedures had the function of a gatekeeper, always letting some people in, letting some people in sometimes, and not letting other people in at all. This confirms previous reports that users do not like to use sites that require them to log in [11]. As of today, this potential gatekeeper effect has received little attention in the medical context, and its clinical significance remains largely unknown. It must be stressed that practitioners and system designers developing Web 2.0 systems should pay attention to the risk of creating a digital divide in their use of interventions that require login procedures. Moreover, the risk of selection bias occurring in studies that require a login procedure is obvious.

Based on respondents’ perceptions of the usefulness of the portal, further development of the portal seems warranted. Specific suggestions from users especially targeted the gatekeeper effect. Thus a largely open portal was suggested in order to facilitate access for all patients and professionals, including school personnel as well as health practitioners. An open portal would also facilitate use by other persons associated with a child or youth with diabetes, such as relatives, friends, and others. It may be that use by young people with diabetes who are somewhat unwilling to identify with the disease would also be enhanced. Experiences related by respondents indicated that an open portal would be more in tune with the view that there are no secrets about having diabetes, and that the disease is no one’s fault. Finally, due to age limits of the pediatric clinics involved and the login required, the portal Diabit during this study targeted selected patients below 19 years of age only. In the future, such resources should target the needs of older adolescents with long-term disease. These young people are in a vulnerable period of establishing their habits and strategies for daily life as an adult living with the disease, and they may also have further experiences to share.

Thus a reasonable approach for practitioners and system designers who develop Web 2.0 systems would be to keep as many functions as possible open, without password requirements, and, when it is necessary to use login procedures, these should be as simple as possible. To enhance openness, participation, and collaboration, we believe that users of the Web 2.0 portal described in the present study should have open access to information supplied by their practitioners and peers, although registration and login may be required to contribute personal comments. Following completion of this study, an open version of the portal was launched.

### Limitations of the Study

Because qualitative methods were used to gain a deeper understanding of the respondents’ reality, it is not possible in this study to make generalizations in a quantitative manner. A mixed methods methodology would allow gathering of both narrative and numeric data, but that implies asking a different research question [41]. Because self-directed essay writing about a topic gives the respondent opportunities to reflect, the two primary questions asked allowed respondents to reflect on positive experiences as well as negative ones. The majority of the respondents were parents; the views of young patients of different ages were not well represented. Hence transferability of the findings should take into consideration the size and characteristics of the sample. Nevertheless, our findings may be similar to what would be found in other populations [42].

### Future Research

Further clinical evaluations from the perspective of young patients’ daily life are needed regarding Web 2.0 environments designed to support coping with chronic disease. More information on needs expressed by young people themselves remains to be obtained, including information about the needs of adolescents at a later stage of the disease. Studies are warranted that would also take into account the views of larger samples of young patients and their families. Interactive systems integrating individual feedback and data monitoring designed for patients with diabetes need further development and evaluation, again, taking into account patients perspectives [28]. Moreover, factors of importance for success and failure need to be identified in patients’ use of social media for their health issues.

### Conclusions

This study suggests that Web 2.0 services have great potential for supporting young patients with type 1 diabetes and their parents, improving their ability to retrieve information, with the goal of enhancing diabetes management. Well-developed Web 2.0 services may contribute to greater use of these services and be more beneficial than initially planned. Such services should be carefully maintained by health care professionals who are “on their toes,” that is, alert and active on the site with new information and updates in the field. When designing Web 2.0 services for young patients and their parents, login procedures should be simplified as much as possible. For the management of chronic diseases, Web publishing, social networking, and other Web 2.0 resources seem to be useful from the patient’s perspective. Practitioners may need education, support, and guidelines to help them use these strategies optimally in collaboration with their patients.
